# *Trans*-cinnamaldehyde–Biosurfactant Complex as a Potent Agent against *Enterococcus faecalis* Biofilms

**DOI:** 10.3390/pharmaceutics14112355

**Published:** 2022-10-31

**Authors:** Mingxin Hu, Shanthini Kalimuthu, Chengfei Zhang, Islam A. A. Ali, Prasanna Neelakantan

**Affiliations:** 1 Faculty of Dentistry, The University of Hong Kong, Hong Kong SAR, China; 2 Department of Endodontics, Faculty of Dentistry, Mansoura University, Mansoura 35516, Egypt

**Keywords:** biofilms, *Enterococcus faecalis*, *trans*-cinnamaldehyde, acidic sophorolipid

## Abstract

*Enterococcus faecalis* is an opportunistic microbial pathogen frequently associated with diverse infections, including those of the skin and teeth, as well as those from surgical wounds. It forms robust biofilms that are highly tolerant to most antimicrobials and first-line antibiotics. Therefore, investigating alternative strategies to eradicate its biofilms is a critical need. We recently demonstrated that *trans*-cinnamaldehyde (TC) potently kills *E. faecalis* biofilm cells and prevents biofilm recovery, and yet, the extreme hydrophobicity of TC hampers clinical translation. Here, we report that a complex of TC with an FDA-approved biosurfactant (acidic sophorolipid/ASL) significantly reduces the bacterial viability and biomass of *E. faecalis* biofilms, compared to TC alone. A confocal laser-scanning microscopic analysis demonstrated that the TC–ASL treatment significantly decreased the biofilm thickness and volume. In conclusion, our study highlights the anti-biofilm potential of the newly developed TC–ASL.

## 1. Introduction

*Enterococcus faecalis* is the most prevalent enterococcal species in critical human infections, such as those of the endocardium, urinary tract, bloodstream, abdomen, and dental root canals as well as those from surgical wounds [1]. Its intrinsic resistance to antibiotics, such as β-lactams and aminoglycosides [2,3], ability to survive under a wide range of hostile conditions [4], and capacity to evade clearance by host immunity [5] contribute to its critical role in such infections. These phenotypes are largely due to its ability to form biofilms on host tissues, medical devices, and catheters [6], wherein resident bacteria of various phenotypes are protected by a hydrated, structurally complex matrix known as an extracellular polymeric substance (EPS) [7]. Therefore, higher doses of antimicrobials are required to eradicate biofilm bacteria, which poses a higher risk of inducing the emergence of antimicrobial resistance (AMR) [8].

Conventional antibiotics have limited antibiofilm effects either due to the inherent tolerance of biofilms or acquired resistance to several antibiotics, which contributes to persistence of *E. faecalis*-associated infections. In fact, mature biofilms have been found to be tolerant to mono- or combinatorial treatments with antibiotics, including ampicillin- and rifampicin-containing combinations [9,10]. Furthermore, *E. faecalis* has demonstrated several drug resistance mechanisms. For instance, the poor penetration through the cell envelope of *E. faecalis* contributes to its resistance to aminoglycosides [11], while the presence of the *lsa* (A) gene that encodes an ABC superfamily of proteins contributes to *E. faecalis*’ resistance to clindamycin [12]. Furthermore, the treatment of *E. faecalis* biofilms with vancomycin was found to induce the expression of cell transport and penicillin-binding proteins involved in β-lactam antibiotics resistance [13]. These findings emphasize the urgent need to identify and explore novel antibiofilm agents against *E. faecalis* with a minimal risk of developing resistance.

The plant kingdom has emerged as a rich reservoir of therapeutics to combat microbial pathogens and suppress their virulent attributes [14]. Phytochemicals are structurally diverse plant-derived secondary metabolites, which demonstrate a wide range of biological activities, including antimicrobial and antibiofilm properties [14,15]. These compounds exert their antimicrobials effects via multiple modes of actions which reduce the likelihood of triggering resistance [16]. Amongst these phytochemicals, *trans*-cinnamaldehyde (TC), a phenylpropanoid which represents the principal constituent of cinnamon essential oil (EO) [17], has been shown to be a broad-spectrum antimicrobial agent [18,19,20]. We have recently demonstrated the antibacterial, antifungal, antibiofilm, and anti-virulence effects of TC [21,22]. However, the biological activity of the phytochemical TC is limited by its lipophilic nature, which reduces its solubility in water-rich environments as encountered in the hydrated EPS matrix [23,24]. Therefore, novel delivery formulations are needed to improve the water solubility and stability of TC, thus maximizing its ant-microbial and antibiofilm properties.

Biosurfactants are a diverse group of FDA-approved, surface-active, environmentally friendly molecules produced by certain fungal or bacterial species [25,26]. Sophorolipids (SLs) are extracellular amphiphilic biosurfactants produced by the non-pathogenic yeast *Starmerella bombicola* [27]. The unique structural configuration, negligible toxicity, and self-assembling properties of SLs to mimic the cell membrane support their application as drug delivery vehicles of bioactive lipophilic compounds [28]. The crude mixture of SLs contains lactonic and acidic forms depending on the arrangement of the constituent fatty acid chains [29]. Acidic sophorolipids (ASLs), which possess a surfactant-like property, consist of a hydrophobic skeleton of oleic acid connected with two hydrophilic ends which contain a sophorose molecule and carboxylic group [30]. We and others have shown that ASLs can be used to encapsulate and improve the antimicrobial, antifungal, and antibiofilm properties of hydrophobic phytochemicals, such as curcumin [28,31,32]. However, the effect of ASLs on the effect of TC on *E. faecalis* biofilms has yet to be explored.

Therefore, the aim of this study was to synthesize a TC–ASL complex and investigate its effect against *E. faecalis* in its planktonic and biofilm (sessile) mode of growth. The hypothesis was that the TC–ASL complex would rapidly kill *E. faecalis* planktonic cells and significantly reduce the bacterial counts, biomass, and biovolume of *E. faecalis* biofilms compared to TC.

## 2. Materials and Methods

### 2.1. Bacterial Strain and Growth Conditions

*E. faecalis* reference strain ATCC 47077 was routinely maintained on horse blood agar. For all the experiments, *E. faecalis* was grown overnight in brain heart infusion broth (BHI) at 37 °C. Before each experiment, the microbial count was adjusted to 2 × 10^6^ colony-forming unit (CFU)/mL. All experiments were performed in triplicate on three independent occasions.

### 2.2. Chemicals

*Trans*-cinnamaldehyde (TC) and dimethyl sulfoxide (DMSO) were purchased from Sigma-Aldrich (St. Louis, MO, USA). ASL was purchased from Biosynth Carbosynth (Compton, Newbury, Berkshire, UK). TC was dissolved in DMSO (5% *v*/*v*) as previously demonstrated by [21]. The 0.2 μm pore syringe filters were used to filter-sterilize all the chemicals before the experiments (Pall Life Sciences, Pensacola, FL, USA).

### 2.3. Synthesis of TC–ASL Complex

To synthesise the TC–ASL complex, probe sonication of different TC concentrations (312.5 to 1250 μg/mL) with 1% ASL was performed using the SONICS^®^ Vibracell-VCX 750 (Sonics & Materials, Inc. Newtown, CT, USA) as described previously [28]. For each cycle, the amplitude was set as 40 Hz, with a constant pulse of 10 s at an interval of 3 s, and this cycle was repeated for 40 min as described previously [31].

### 2.4. Scanning Electron Microscopy (SEM) and Transmission Electron Microscopy (TEM) of TC–ASL

To characterize the size and morphology of TC–ASL, scanning electron microscopy (SEM) of TC–ASL and ASL were obtained from Hitachi VP-SEM SU1510 (SU-1510, HITACHI, Minato-ku, Tokyo, Japan) at voltage 15 kV and transmission electron microscopy (TEM) images of TC–ASL were obtained from Philips CM100 (Philips, Eindhoven, The Netherlands), respectively. For SEM analysis, 10 μL of non-diluted samples was drop-cast on a coverslip and air-dried overnight followed by being sputter coated with palladium and platinum [28]. For TEM analysis, about 10 μL of samples diluted 100 times in deionized water was drop-cast on a carbon-coated copper grid (mesh size 200) and air-dried overnight [28]. Image J was used to measure the size of TC–ASL micelles.

### 2.5. Time-Kill Kinetics and Membrane Disruption

The effect of TC–ASL, TC, and ASL on the growth of the *E. faecalis* was assessed. Briefly, the cell suspensions of *E. faecalis* was prepared as mentioned above and treated with varying concentrations of TC, TC–ASL, and 1% ASL. The well plates were then incubated at 37 °C for 18 h and the absorbance was measured every hour. Vehicle controls containing DMSO and sterility controls with media alone were routinely maintained for all the trials. The experiments were performed in triplicate on three independent occasions.

To assess the membrane disruptive ability of TC–ASL, TC, and 1% ASL, the planktonic cell suspension was treated with varying concentrations of TC–ASL, TC, and 1% ASL as described above. Then, 20 μL of sample was collected after 4 h and placed on sterile coverslips, air-dried, and fixed overnight with 2.5% glutaraldehyde at 4 °C. After fixation, the samples were dehydrated in a series of ethanol solutions (75%, 80%, 95%, and 100% ethanol for 10–15 min). After gold sputter-coating, the slides were examined using a scanning electron microscope at 15 K magnification (SU1510, HITACHI, Minato-ku, Tokyo, Japan) to characterize the bacterial membrane integrity.

### 2.6. Biofilm Formation and Treatment Assay

*E. faecalis* biofilms were developed by inoculating the wells of sterile 96-well polystyrene plates with 200 μL of 10^6^ CFU/mL aliquots of the prepared inoculum suspended in BHI medium. The plates were incubated at 37 °C for 72 h under aerobic conditions with daily replenishment of fresh BHI [21]. After the incubation period, the supernatant was carefully removed, and the biofilms were gently washed thrice with PBS to remove the non-adherent cells. The biofilms were then treated with TC–ASL, TC, and ASL for 24 h. The final TC concentrations in TC–ASL, TC, and ASL treatment groups were 312.5, 625, and 1250 μg/mL. Biofilms incubated with deionized water were considered to be a control. Our preliminary studies confirmed that the DMSO vehicle had no effect on the bacteria. The treatment solutions were then aspirated, and the treated biofilms were gently washed thrice with PBS and used for further analyses as described below.

### 2.7. Quantification of Viable Biofilm Cell Count

The colony-forming unit (CFU) assay was used to quantify the viable biofilm cells after treatment. Briefly, the biofilm cells were collected by vigorous scrapping and pipetting. The collected biofilm suspensions were serially diluted in PBS then plated on blood agar and incubated at 37 °C for 24 h. The number of viable cells was determined by counting the CFUs, which were transformed into log_10_ values.

### 2.8. Biomass Quantification

The overall biomass of *E. faecalis* biofilm was evaluated using the safranin assay [33]. Two hundred microliters of 0.1% safranin solution (Safranin O, Sigma-Aldrich, Burlington, MA, USA) was used to stain the treated biofilms for 30 min at room temperature. The stained biofilms were then rinsed with sterile PBS to remove the excess dye and left to dry for 15 min. The retained dye was solubilized with 33% acetic acid for 15 min. The supernatants (100 µL) in each well were then transferred to a new 96-well plate and the absorbance was measured at 492 nm using a spectrophotometer (SpectraMax M2, Molecular Devices, Sunnyvale, CA, USA).

### 2.9. Confocal Laser-Scanning Microscopic (CLSM) Analysis

The treated biofilms in 8-well chamber slides (ibidi GmbH, Grafelfing, Germany) were stained using BacLight Bacterial Viability Kit^TM^ (L7007, Thermo Fisher Scientific, Waltham, MA, USA). The slides were then incubated in dark for 30 min following the manufacturer’s instructions. The stained biofilms were visualized using an oil-immersion objective lens (×60) of a confocal laser-scanning microscope (CLSM; Fluoview FV 1000, Olympus, Tokyo, Japan) at three randomly selected points. The biofilm z-stacks were reconstructed into 3D images, which were analyzed using IMARIS software (Bitplane, St. Paul, MN, USA) to quantify the biofilm thickness and volume.

### 2.10. Statistical Analysis

One-way ANOVA with post hoc Tukey HSD test was used to compare the results between the experimental groups. A *p*-value ≤ 0.05 was considered statistically significant. Statistical analyses was performed using the SPSS software version 23 (IBM, Armonk, NY, USA).

## 3. Results

### 3.1. TC–ASL Forms Spherical to Ellipsoidal Micelles

**Figure 1:**
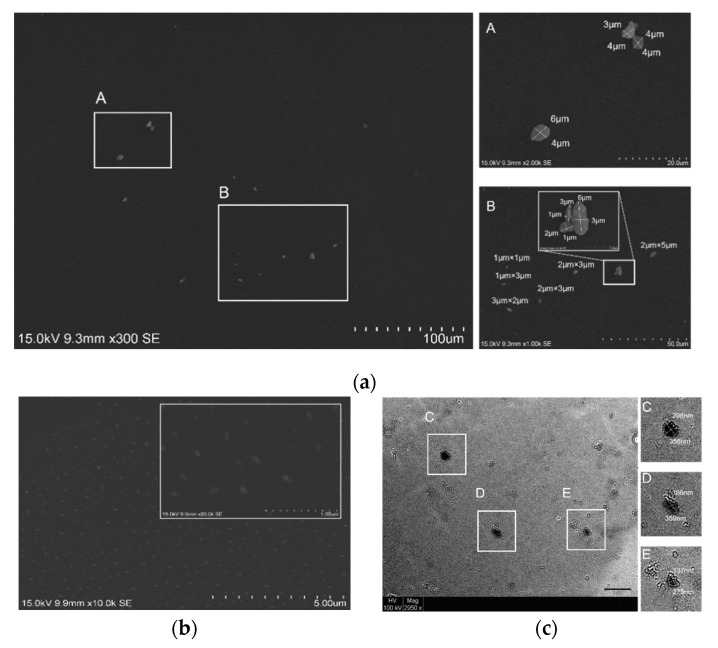
(**a**) SEM image (300×) of ASL micelles. Inset A and B show the magnified images (1k× & 2k×) of ASL micelles with size measurements by Image J. The inset in B showed a magnified image (10k×) of an ASL micelle. (**b**) SEM image (10k×) of TC–ASL micelles. The inset showed a magnified image (50k×) of TC–ASL micelles. (**c**) TEM image (2950×) of TC–ASL micelles. Inset C, D, and E show the magnified images of TC–ASL micelles with size measurements by Image J.

The SEM examination demonstrated that the ASL micelles were approximately 1–6 μm in size with spherical and elliptical shapes (Figure 1a). Evenly formed micelles of TC–ASL with spherical and ellipsoidal morphology were observed from the SEM (Figure 1b) analysis. Further analyses with TEM confirmed the spherical and ellipsoidal micelles formed due to physical sonication forces with sizes ranging from 135–360 nm (Figure 1c).

### 3.2. TC–ASL Complex Rapidly Arrests Planktonic Bacterial Growth and Disrupts the Bacterial Cell Membrane

Our pilot study demonstrated that the minimum inhibitory concentration (MIC) of TC against *E. faecalis* ATCC 47077 was 625 μg/mL. Based on this finding and those from our previous work [21], we first examined the ability of 10 mg/mL to 78.1 μg/mL in the presence or absence of 1% ASL to significantly reduce the cell viability of *E. faecalis* biofilms. Based on the results of this pilot study, MIC/2, MIC, and 2MIC were chosen for further experiments. Equivalent concentrations of TC and 1% ASL were also tested in all the experiments.

**Figure 2:**
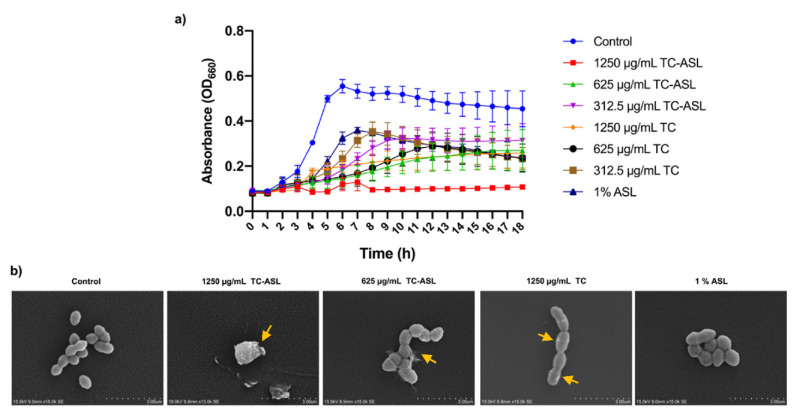
Time–kill kinetics of TC–ASL, TC, and ASL against planktonic *E. faecalis*. (**a**) 2 × 10^6^ CFU/mL of planktonic *E. faecalis* was treated with 1250, 625, and 312.5 μg/mL of TC, the respective concentrations of TC mixed with 1% ASL, or pristine 1% ASL for 18 h at 37 °C. At each hour, the absorbance was recorded, and the graph depicts the time-dependent effects of these treatment groups against *E. faecalis*. (**b**) Membrane-disruptive ability: SEM images show the loss of bacterial membrane integrity and shrinkage of the cells in the 1250, 625 μg/mL TC–ASL groups, whereas intact membrane was observed in the control, Pristine TC, and ASL alone groups. This indicates that the mechanism of action of TC–ASL to kill the cells is membrane disruption. The yellow arrows indicate changes in the bacterial cell membrane following treatment.

The absorbance (OD_660nm_) vs. time graph shows that 1250 μg/mL of TC–ASL rapidly arrested bacterial growth at just 1 h of treatment and this was maintained throughout the assessment period of 18 h, when compared to the pristine TC and ASL groups (Figure 2a). When compared to the control, 1250 μg/mL of TC–ASL showed more than a 90% growth inhibition after 18 h of incubation, indicating superior antibacterial effects when compared to the other groups.

To decipher the mechanism of the antibacterial activity of TC–ASL, the integrity of the cell membrane was assessed using SEM. Even within 4 h of treatment, TC–ASL completely disrupted the bacterial cell membrane, eventually leading to the leakage of the intracellular contents followed by cell death. On the other hand, the membrane integrity showed minimal changes in the TC-treated groups while the cell membrane was intact in the ASL-treated bacteria. These findings indicate that greater concentrations of TC and ASL may be required to disrupt the bacterial cell membrane.

### 3.3. TC–ASL Complex Kills E. faecalis Biofilm Cells

**Figure 3:**
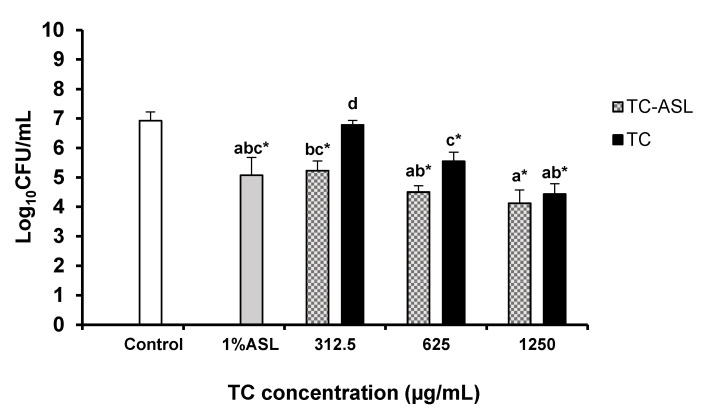
Cell count of *E. faecalis* biofilm after treatment with TC–ASL, TC, and 1% ASL. The cell count was expressed in log_10_ CFU/mL. The results represent the mean ± SD of three replicates in independent experiments. Different letters denote a statistically significant difference (*p* ≤ 0.05) between different treatment groups while the same letters show no statistical difference (*p* > 0.05) between different treatment groups, including the within-group comparison and between-group comparison. * Denotes a significant difference compared to the control (*p* ≤ 0.05).

A significant dose-dependent reduction in log_10_ CFU/mL was observed in the TC–ASL treatment groups compared to the control (*p* < 0.001, Figure 3). TC–ASL reduced viable biofilm cells by 1.7–2.8 log_10_ CFU/mL. A similar pattern was shown in the TC-treated biofilms, wherein viable cells were reduced by 0.15, 1.38, and 2.49 log_10_ CFU/mL at 312.5, 625, and 1250 μg/mL, respectively. However, this reduction was only significant at 625 and 1250 μg/mL (*p* < 0.01 and *p* < 0.001, respectively, Figure 3). Treatment with TC–ASL resulted in significantly lower log_10_ CFU/mL compared to TC alone, and the final TC concentration was 312.5 μg/mL and 625 μg/mL (*p* = 0.002 and *p* = 0.046, Figure 3). ASL, on the other hand, demonstrated a significant reduction in viable biofilm cells (*p* < 0.001, Figure 3) compared to the control.

### 3.4. TC–ASL Complex Reduces the Biomass of E. faecalis Biofilm

**Figure 4:**
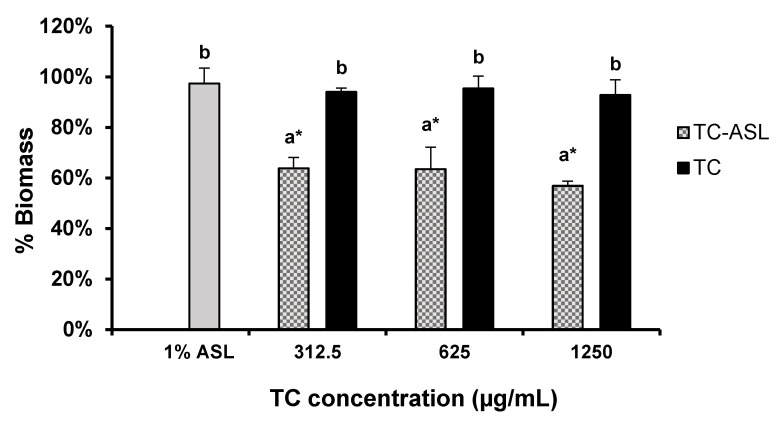
Biomass of *E. faecalis* biofilm after treatment with TC–ASL, TC, and ASL. The biomass of treated biofilm was expressed relative to control (100%). The results represent the mean ± SD of three replicates in independent experiments. Different letters denote a statistically significant difference (*p* ≤ 0.05) between different treatment groups while the same letters show no statistical difference (*p* > 0.05) between different treatment groups, including the within-group comparison and between-group comparison. * Denotes a significant difference compared to the control (*p* ≤ 0.05).

TC–ASL significantly reduced the biofilm biomass compared to the control (*p* < 0.001, Figure 4). On the other hand, the reduction in the biofilm biomass was insignificant in the TC (5–7%) and ASL (3%) treatment groups compared to the control (*p* > 0.5, Figure 4). In contrast, the biomass of *E. faecalis* biofilms was reduced by 36–43% in the TC–ASL treated groups, significantly improving the biomass reduction in comparison to TC (*p* < 0.001, Figure 4).

### 3.5. TC–ASL Reduces Biofilm Thickness and Alters the Biofilm Architecture

**Figure 5:**
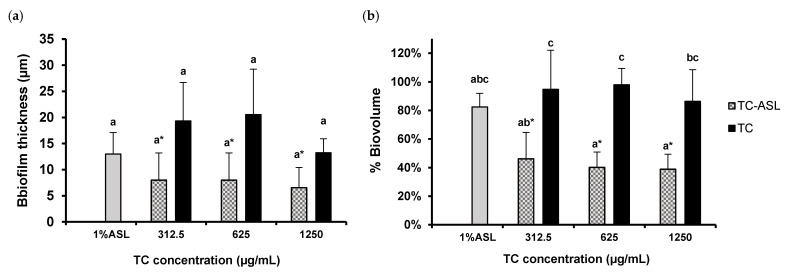
Quantification of (**a**) biofilm thickness or (**b**) biofilm volume after treatment with TC–ASL, TC, and 1% ASL. The biofilm thickness was obtained from the z-stacks while the volume of treated biofilm was expressed relative to the control (sterile-water-treated biofilm)(100%). The results represent the average of three independent experiments ± SD. Different letters denote a statistically significant difference (*p* ≤ 0.05) between different treatment groups while the same letters show no statistical difference (*p* > 0.05) between different treatment groups, including the within-group comparison and between-group comparison. * Denotes a significant difference compared to the control (*p* ≤ 0.05).

The structural properties of the control and treated biofilms were described in terms of biofilm thickness and biovolume (Figure 5). The biofilm thickness decreased by 70 ± 22% in the 312.5 and 625 μg/mL TC–ASL treatment groups and by 75 ± 16% in the 1250 μg/mL TC–ASL group relative to the control, respectively (Figure 5a). However, the biofilm morphology remained slightly altered or unperturbed when treated with TC or ASL alone. While the percentage of the reductions in biofilm thickness ranged from 24 ± 33% to 53 ± 14% for these groups, there was a significant reduction in biovolume after the treatment with TC–ASL (*p* < 0.05, Figure 5b) compared to the control. The biovolume after the TC–ASL treatment groups was significantly lower compared to those in the TC-treated groups (*p* < 0.05, Figure 5b).

**Figure 6:**
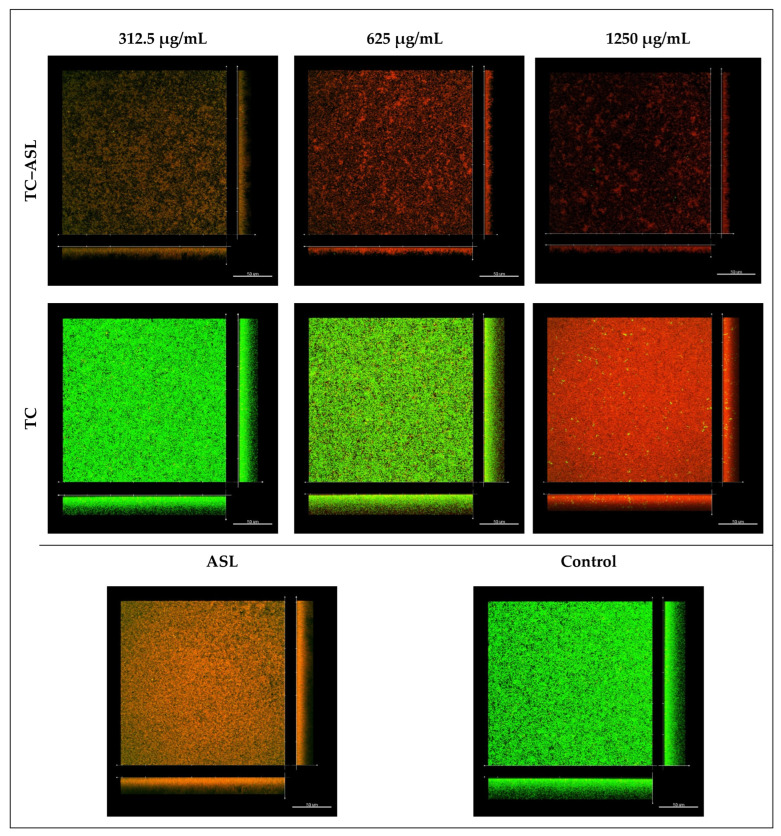
Confocal microscopic images (×60) of *E. faecalis* biofilms after treatment of TC–ASL, TC, and 1% ASL. The cells with the intact membrane (live) are labeled with the green fluorescent dye (STYO-9), and the cells with damaged membranes (dead) are labeled with red fluorescent dye (PI). The partially damaged cell membranes in which the STYO-9/PI was retained within the cells showed a yellowish-green color [34].

The biofilm structure after different treatments was visualized by CLSM. The control group showed a thick and densely packed biofilm with apparently closely aggregated live cell clusters (green fluorescence) (Figure 6), indicating an intact bacterial cell membrane and undisturbed biofilms. Similar densely packed microcolonies were observed in biofilms treated with TC. A dose-dependent increase in the dead cell population was noted in TC–ASL-treated biofilms as indicated by the fact that more red-stained bacteria were viewed at higher concentrations (Figure 6).

The percentage of dead cells was calculated as the average number of dead cells divided by the average number of total cells in the biofilms of each group. There was a significant difference in the percentages of dead cell between the biofilms treated with 1250 µg/mL TC (52 ± 2.5%) and the control group (36 ± 1.8%) (*p* < 0.001). In contrast, treatment with TC–ASL disrupted the biofilm’s architecture, leaving only isolated microcolonies with a sparsely distributed, minor population of green-stained bacterial cells (Figure 6). The percentage of dead bacteria was significantly increased in the TC–ASL-treated biofilms compared to that of the control group (*p* < 0.05). The percentage of dead cells in the TC–ASL-treated biofilms (51 ± 0.7% and 52 ± 1.1%) at 312.5 and 625 μg/mL were significantly higher than those observed in the corresponding TC concentrations (36 ± 3.9% and 40 ± 1.8%) (*p* < 0.001). The structure of biofilms treated with ASL alone remained relatively unaffected.

## 4. Discussion

*E. faecalis* is a biofilm-forming pathogen known for its tolerance to several antibiotics and disinfectants once its biofilm has been established [10]. Therefore, a novel strategy to eradicate its biofilm cells and disrupt the biofilm structure should be investigated. In the current study, we used the ATCC 47077 strain, which is derived from a human caries-associated strain [35] and is frequently used in laboratory studies [36,37,38].

TC is the major active component responsible for the antimicrobial activity in cinnamon essential oils [39]. We previously reported that 0.5% to 1% of TC significantly reduced the viability of *E. faecalis* biofilm cells with limited changes in biofilm structures and exopolysaccharide contents, indicating the necessity of adjuvants to potentiate its activity as an antibiofilm agent and of tackling problems due to its extreme hydrophobicity [21]. A study on a curcumin–sophorolipid complex demonstrated that 1–5% *w*/*v* ASL could encapsulate curcumin and significantly improved the antibiofilm activity of curcumin [28]. Therefore, it is reasonable to assume that ASL within this range of concentrations may also encapsulate the TC and boost its antibiofilm effect. Our preliminary experiments based on a previous study [21] confirmed that 1% ASL combined with a lower concentration (312.5 μg/mL, 625 μg/mL, 1250 μg/mL) of TC significantly reduced the biofilm biomass. Hence, these three concentrations of TC were used to further investigate the antimicrobial and antibiofilm effect of TC–ASL.

This study reports the synthesis as well as the antimicrobial and antibiofilm effects of TC–ASL for the first time. The synthesis was based on other well-established protocols [28,31,32]. The self-assembling ASL micelles could be divided into three domains: the hydrophilic shell, the aliphatic core, and the intermediate palisade layer [40]. It is speculated that the hydrophobic TC may be anchored within the palisade layer of ASL micelles by its hydrophobic skeleton [28], while the hydrophilic shell of the micelles could be attached to the hydrated EPS matrix [41,42,43], thus enhancing the interaction between TC and biofilm bacteria.

Our results revealed a dose-dependent and significant reduction in viable cell counts in the TC–ASL (312.5, 625, and 1250 μg/mL) and TC (625 and 1250 μg/mL) groups compared to the control group. This agreed with the result reported by Ali et al. [21], wherein TC reduced the viability of mature *E. faecalis* biofilms. A significant biomass reduction was observed in the TC–ASL groups but not in the TC groups, given the limited ability of TC to disrupt the biofilm structure [21].

It was interesting to note that the biofilm viable cell counts after the 1% ASL treatment were significantly reduced without affecting the overall biomass, suggesting a limited antibiofilm activity. Although ASL is frequently regarded as a drug-delivery carrier, its antimicrobial activities against *E. faecalis* biofilm were reported previously, showing that 0.5% *w*/*v* ASL significantly reduced the viable cell counts in *E. faecalis* biofilm after 12 h [29]. This could be attributed to destabilization and increased permeability of the bacterial cell membrane by ASL [39,44,45].

Our results revealed the limited ability of TC or ASL to induce significant biofilm destruction, as revealed by the biomass quantification and CLSM assays. Our results differ from those of De Rienzo et al., who showed that SLs disrupted biofilms of other Gram-positive bacteria, such as *Staphylococcus aureus* and *Bacillus subtilis* [46]. Their study used a higher concentration of SL (5%) to treat biofilms that were 24 h old. Differences in the SL concentration, test microorganism, and biofilm age could account for the differences in the results. When combined with ASL, TC was found to be more effective in reducing biofilm viability and biomass compared to TC alone. As described earlier, the ASL helped to establish the micellar structure, which encapsulates the TC molecule in its core, while the exposed hydrophilic tails of ASL on the outer surface enhanced the penetration of TC into the EPS matrix of *E. faecalis* biofilm, thus improving the antibiofilm activity of TC [28,31]. It is also possible that ASL could span through cell membranes, thus improving the intracellular infiltration of antimicrobial agents [47], which leads to a significant disruption in bacterial physiology.

Our results showed a significant reduction in biofilm viability between the TC–ASL and TC treatment groups at lower concentrations (312.5 and 625 μg/mL). However, this reduction was not significant at the higher concentration. This could be related to the finding that 1250 μg/mL TC reduced the viable cells by 2.49 (≃2.5) log_10_ CFU/mL, which is equivalent to killing more than 90% of the biofilm cells; thus, an insignificant reduction in biofilm viable cells upon the treatment with TC–ASL at this concentration was expected. Furthermore, the difference in the biomass reduction was statistically significant between TC–ASL and TC. This confirms the role of ASL in potentiating the effect of TC against the overall biomass of *E. faecalis* biofilms. However, this reduction was not significantly improved by increasing the final TC concentration in the TC–ASL complex. A plausible explanation was that 1% ASL might be insufficient to encapsulate all the TC molecules at a higher concentration [28], resulting in an insignificant biomass reduction within TC–ASL-treated biofilms.

The cell viability and structural changes in *E. faecalis* biofilm were visualized by CLSM by using a fluorescent live/dead viability assay. A disrupted architecture with sparse biofilm cells and isolated cell aggregates were found in biofilms treated with TC–ASL. This was different from the undisrupted biofilm architecture in the TC and control groups. It may be hypothesized that the TC–ASL micelles formed voids or channels inside the biofilm structure, facilitating a closer interaction between the treatment agents and the biofilm cells [32]. This could be further augmented by the surface-tension-lowering property of biosurfactants, which improves the penetration of TC into the biofilm structure. Future works should focus on determining the effectiveness of TC–ASL against polymicrobial biofilms, dissect the mechanism of the antibiofilm activity of TC–ASL, and confirm the compatibility of TC–ASL with mammalian cells. The ability of TC–ASL to inhibit biofilm formation and suppress post-treatment biofilm growth should be also considered in future investigations.

## 5. Conclusions

This study demonstrated for the first time that *trans*-cinnamaldehyde combined with acidic sophorolipids showed significantly improved antibiofilm effects by improving bacterial killing, reducing the biomass and volume of *E. faecalis* biofilm compared to *trans*-cinnamaldehyde alone.

## Data Availability

The data are available through a personal request to the corresponding authors.
